# GTPBP2 positively regulates the invasion, migration and proliferation of non-small cell lung cancer

**DOI:** 10.7150/jca.48340

**Published:** 2021-05-05

**Authors:** Liu Jie, Li Cong, Wei Conghui, Gao Ying

**Affiliations:** 1 Department of Pathology, The Fourth Hospital Affiliated to China Medical University, Shenyang, Liaoning. 110032 People's Republic of China.; 2 Department of Pathology, The First Affiliated Hospital of Anhui Medical University, Jixi street, Hefei, Anhui. 230000 People's Republic of China.; 3 Department of Pathology, The Second Affiliated Hospital of Nanchang University, Nanchang, Jiangxi, 330000. People's Republic of China.

**Keywords:** GTPBP2, cell proliferation, cell invasion, cell migration, non-small cell lung cancer.

## Abstract

Lung cancer is one of the most common malignant tumors in the world, and the mortality rate ranks first among various malignant tumors. GTP-binding proteins (guanosine 5'-triphosphate-binding proteins, GTPBPs) are a type of protein with signal transduction function, have GTP hydrolase activity, and play an important role in cell signal transmission, cytoskeletal regulation, protein synthesis and other activities. GTPBP2 is one of the members of the G protein superfamily. Research on GTPBP2 is currently focused on human genetics, and its research in tumors has not been reported. First, Western blot and quantitative real-time PCR were used to analyze the expression differences of 12 cases of GTPBP2 in human NSCLC fresh cancer tissues and adjacent tissues. Then we selected 112 cases of NSCLC cancer tissues and 65 adjacent tissues for immunohistochemistry experiments to analyze the relationships between the expression of GTPBP2 and clinical pathological parameters and prognosis, we found that GTPBP2 is highly expressed in NSCLC cancer tissues, and the high expression of GTPBP2 is related to pTNM stage and lymph node metastasis. In addition, after GTPBP2 knockdown, GTPBP2 can promote the proliferation and invasion of NSCLC cell lines by up-regulating RhoC and MMP-9, and up-regulate cyclinD1, CDK4 and c-myc, and down-regulate P27 to promote the invasion of NSCLC cell lines. In addition, GTPBP2 negatively regulates Axin to promote β-catenin expression, thereby activating Wnt/β-catenin signaling, and promoting the occurrence of NSCLC.

## 1. Introduction

As one of the most common malignant tumors in the world, lung cancer ranks first among all types of malignant tumors. GTP-binding proteins (guanosine 5'-triphosphate-binding proteins, GTPBPs) are a type of protein with signal transduction function, have GTP hydrolase activity, and play an important role in cell signal transmission, cytoskeletal regulation, protein synthesis and other activities. GTPBP2 is one of the members of the G protein superfamily, which can interact with the components of the signal transduction pathway and may be involved in the development of tumors.

The new reports show that in Xenopus laevis, GTPBP2 was injected into embryos after antisense morpholine oligonucleotide knockdown (Morpholino), and the mRNA levels of Axin were found to increase 1. Under normal circumstances, Axin can form a complex with GSK-3β, APC, β-catenin, etc. Phosphorylating β-catenin and down-regulating its level, thereby negatively regulating the Wnt/β-catenin signaling pathway, participating in cell growth and development, and inhibiting the occurrence and development of tumors. When the level of Axin increases abnormally, β-catenin cannot be degraded, and it accumulates abnormally in the nucleus, acting on transcriptional activators, causing malignant transformation of tumors. The decrease of GTPBP2 level can increase the expression level of Axin, therefore, GTPBP2 may be a positive regulator of Wnt/β-catenin signaling pathway.

At present, the relationship between the expression of GTPBP2 gene and tumors has not been reported in domestic and foreign literatures, and the expression in human lung cancer has not been studied.

## 2. Materials and methods

### 2.1. Patients and samples

Collected 112 cases of non-small cell lung cancer tissues and 65 cases of normal lung tissues that were surgically removed in the Fourth Affiliated Hospital of China Medical University from January 2005 to December 2008, and collected operations from January 2018 to October 2018, the 12 pairs of fresh non-small cell lung cancer tissues and normal tissues were removed. None of the enrolled patients underwent radiotherapy and chemotherapy before and after surgery. This study was supported by the Medical Research Ethics Committee of China Medical University, and all patients signed an informed consent.

### 2.2. Cell culture and treatment

A549, H460, H1299, SK-MES-1, H1975, HCC827, 16HBE cells were purchased from Shanghai Cell Bank, Chinese Academy of Sciences. A549, H460, H1299, H1975, HCC827 were cultured in the incubator with 1640 culture medium containing 10% BI fetal bovine serum. SK-MES-1 and 16HBE cells were cultured in the incubator with MEM culture medium containing 10% BI fetal bovine serum , The culture conditions are 37°C, 5% CO2.

A549 cells (Hanheng Biotechnology Co., Ltd., China) were transfected with lentivirus-mediated GTPBP2 virus. This vector contains PURO resistance gene, carries GFP gene, and expresses green fluorescent protein.

### 2.3. Immunohistochemistry

All surgically removed specimens were fixed in 10% neutral formalin, sliced ​​at a thickness of 4 μm, the slices were placed in xylene to dewax and hydrated with gradient alcohol. High-pressure antigen repair was performed in citrate buffer for 2 min, and GTPBP2 rabbit anti-human polyclonal antibody (1: 100 dilution, Biorbyt, UK) was incubated overnight at 4°C. Incubate the secondary antibody, add DAB for color development, counterstain with hematoxylin, dehydrate in gradient alcohol before sectioning and mounting.

### 2.4. Quantitative real-time PCR

Quantitative real-time PCR was performed in a 7500 PCR instrument (Applied Biosystems 7500, USA), 20 l system, following the cycle conditions: pre-denaturation 95°C 30s, PCR reaction 95°C, 5s, 60°C, 34s, using 2^-△△CT^ method to calculate the experimental data CT value, and use graphpad prism5 to analyze the results. The gene encoded by GAPDH is used as an internal reference and the sequence is as follows: GTPBP2, FP: CTGGCTGAGGAGGAAATG; GTPBP2, RP: CACACGGAGGTCTAGGAAC.GAPDH, FP: GAAGGTGAAGGTCGGAGT, GAPDH RP: GAAGATGGTGATGGGATTTC. All experiments were repeated at least 3 times.

### 2.5. Western Blot

NP-40 lysed tissues and cells, lysed on ultrasonic cell crusher and centrifuged by ultracentrifuge to obtain protein, protein concentration was measured by BCA method, electrophoresis of protein (30 μg/well) in SDS-PAGE electrophoresis solution (concentration 10% gel), PVDF The membrane (Millpore, USA) was transferred to the membrane and incubated with the corresponding antibody (Table 1) at 4°C overnight, and then incubated with HRP-labeled anti-rabbit/mouse IgG for 2 hours at room temperature, with ECL detects luminescence. Antibodies used for western blotting in this paper show in Table 1.

### 2.6. Cell proliferation experiment

The cell proliferation experiment was carried out in a 96-well plate, with 2500 cells per well and 100μl of culture medium per well. After 4h, the cells were all submerged to the bottom of the plate and 10μl of CCK8 solution (Biyuntian, China) was added to each well. OD value, record the OD value of the cell at 0h at this time, and measure the OD value at 24h, 48h and 72h respectively, and analyze the obtained data for curve analysis. All experimental results were repeated individually at least 3 times under the same conditions.

### 2.7. Cell migration

The cell migration experiment was conducted in a six-well plate. Three straight lines were drawn on the back of the six-well plate in parallel with a ruler in parallel, 4x10^5 cells per well, 2ml of 1640 complete medium per well, and placed in a 5% CO2 incubator overnight. Use a 200μl pipette tip to draw a line perpendicular to three parallel lines, wash the floating cells with PBS, discard the original medium, add 1640 medium containing 2% serum, observe under the microscope and take a picture, then record it as 0h, then the cell healing area of ​​the shRNA-GTPBP2 group and shRNA-NC group was recorded after 24h and 48h. ImageJ-32 was used to calculate the healing area, and Graphpad prism5 was used to plot, analyze and compare the experimental results.

### 2.8. Invasion experiment

Cell invasion experiments were performed in a 24-well transwell chamber (Corning, USA). For the cell invasion experiment, each chamber was covered with 100ul of Matrigel (1: 9 dilution, BD Biosciences), and incubated at 37°C overnight. The transfected cells were digested with trypsin and counted. 200μl of serum-free medium cell suspension, 600μl of medium containing 20% ​​FBS was added to the lower chamber. Incubate at 37 ° C for 24h. Take out the upper chamber, wash with PBS (preheating), wash twice with PBS, fix with methanol for 20 min, wash with PBS twice after removing methanol completely. Add crystal violet (0.1%) for staining, 20-30min at room temperature, wash twice with PBS, wipe the upper surface cells with a cotton ball, randomly select 10 fields of view under a microscope at high power to take pictures, count, and take the average value for statistics. All experimental results were repeated individually at least 3 times under the same conditions.

### 2.9. Data analysis

The data was analyzed with Spss16.0 software. The survival of NSCLC patients was analyzed by Kaplan-Meier. The relationship between the expression of GTPBP2 and the clinicopathological parameters passed the chi-square test, and the difference between the cells was passed the t test. The P value <0.05 was significant.

## 3. Results

### 3.1. The expression of GTPBP2 after knockdown has important clinical significance

**Figure 1:**
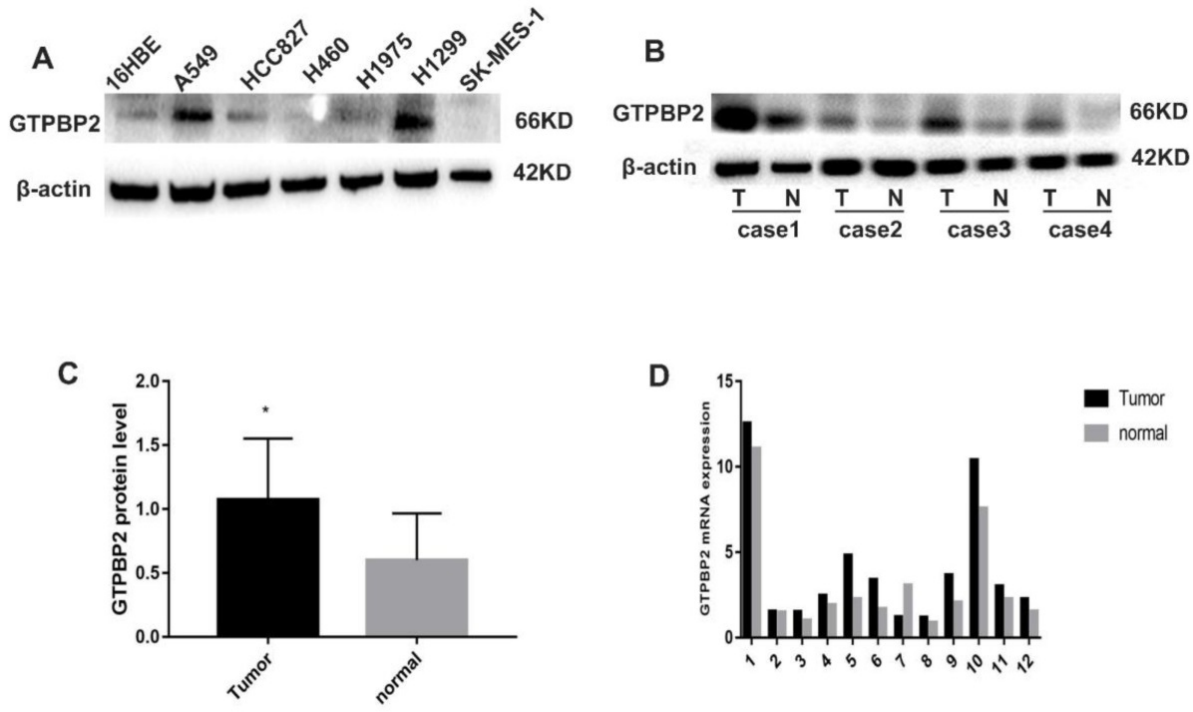
Expression pattern of GTPBP2. A, GTPBP2 expression in non-small lung cancer (NSCLC) cell lines with a normal bronchial cell line (16HBE). B and C, GTPBP2 expression in non-small lung cancer (NSCLC) cancer tissues and normal tissues, analyzed by immunocytochemistry, and assessed by ImageJ software. D, GTPBP2 expression in non-small lung cancer (NSCLC) cancer tissues and normal tissues, analyzed by quantitative real-time PCR expression (P<0.05).

Compared with normal bronchial epithelial cells 16HBE, the expression of GTPBP2 protein in NSCLC cell lines A549, H460, H1299, SK-MES-1, H1975, HCC827 differed in 16HBE expression (Figure 1A). The results showed that the expression of GTPBP2 protein in A549 and H1299 cells expressed higher, compared with other cell lines, and the lowest is 16HBE, so we chose A549 for follow-up experiments.

**Figure 2:**
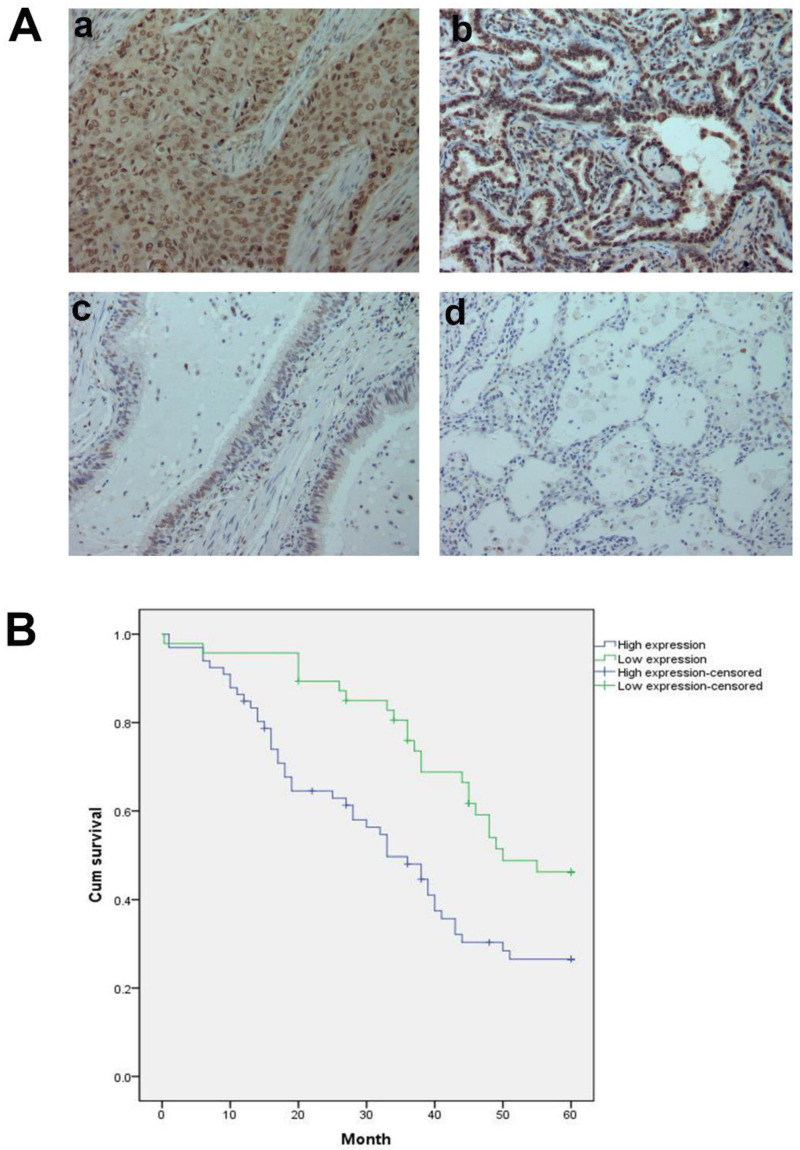
A, GTPBP2 protein expression analyzed by immunocytochemistry in (a) squamous cell carcinoma and (b)adenocarcinoma, with (c)normal bronchial and (d)alveolar epithelial cells. Magnificatin,X200. B, Survival of NSCLC patients with high and low GTPBP2 expression analyzed based on Kaplan-Meier Curves (P<0.05).

In addition, we analyzed 12 cases of fresh lung cancer tissues and corresponding normal tissues by WB. We found that the expression of GTPBP2 protein in fresh lung cancer tissues was different from that in normal tissues (Figure 1B), and the expression level in fresh lung cancer tissues was higher than that in normal tissues (Figure 1C). We also confirmed at the mRNA level by quantitative real-time PCR (Figure 1D). The expression of GTPBP2 protein was also confirmed by immunohistochemistry in 112 cancer tissues and 65 adjacent normal lung tissues. GTPBP2 had a stronger staining depth in cancer tissues than in adjacent tissues (Figure 2A).

At the same time, we analyzed the relationship between the protein expression of GTPBP2 and the pathological parameters of patients (Table 2). The high expression of GTPBP2 was positively correlated with the late stage of TNM stage (P = 0.005 <0.05), and was also positively correlated with lymph node metastasis (P = 0.014 <0.05), not related to age, gender, histological type and degree of differentiation. Kaplan-Meier analysis showed that the high expression of GTPBP2 showed a higher 5-year survival rate and lower expression rate after operation, with a statistically significant difference (Figure 2B).

### 3.2. GTPBP2 promotes the migration, invasion and proliferation of NSCLC cell lines

**Figure 3:**
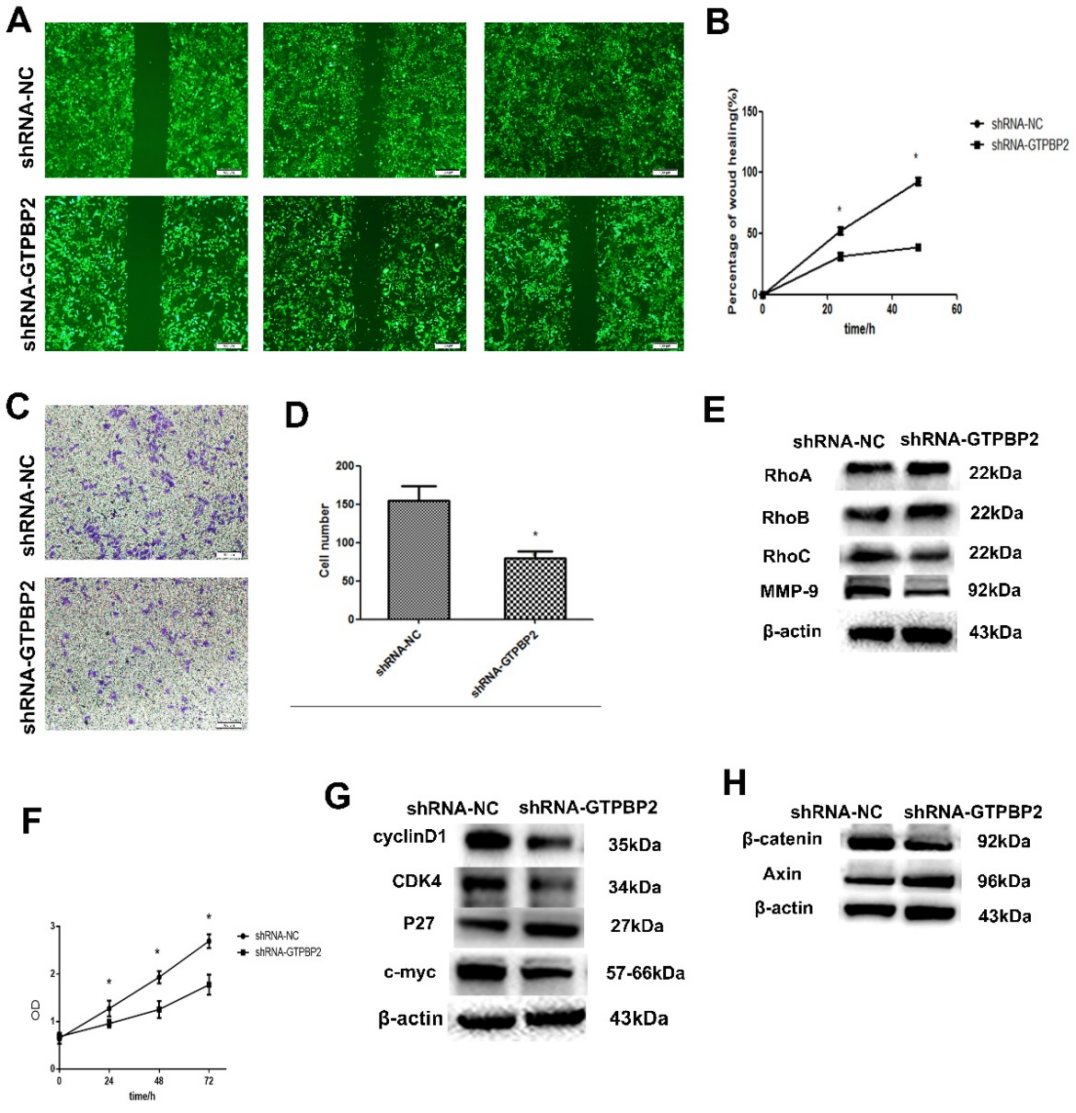
Effect of GTPBP2 expression on the migration and invasion of non-small cell lung cancer (NSCLC) cells. A549 was transfected with specific shRNA-GTPBP2 and shRNA-NC. A and B, the cell migration analyzed by scratch test, and analyzed by Graphpad prism5(shRNA-GTPBP2 vs shRNA-NC, P<0.05). C and D, the cell invasion analyzed by transwell test, and analyzed by ImageJ-32(shRNA-GTPBP2 vs shRNA-NC, P<0.05). E, Expression of GTPBP2 in cell migration and invasion related proteins in A549 cell. F, Cell proliferation analyzed by CCK8, and analyzed by Graphpad prism5(shRNA-GTPBP2 vs shRNA-NC, P<0.05). G and H, the effect of GTPBP2 expression on cell proliferation and Wnt/β-catenin signaling pathway related proteins. (shRNA-GTPBP2 vs shRNA-NC, P<0.05).

Since the expression of GTPBP2 is closed related to the NSCLC process, we further explored the biological function of NSCLC. Through shRNA-mediated GTPBP2 knockdown, we found that the decrease in GTPBP2 expression level reduced the migration and invasion function of the A549 cell line (Figure 3A, B, C, D). To this end, we further examined the corresponding protein expression. We found that after GTPBP2 knockdown, there was no significant change in cell migration-related proteins RhoA and RhoB, while the expression of RhoC was reduced, and cell invasion-related protein MMP-9 expression was reduced (Figure 3E). It shows that the change of GTPBP2 may affect the invasion and migration of cells. Similarly, we found that knockdown of GTPBP2 resulted in decreased cell proliferation capacity (Figure 3F), decreased expression of cell proliferation-related proteins cyclinD1, CDK4 and c-myc, and increased expression of P27 (Figure 3G), indicating that changes in GTPBP2 may affect cells of proliferation.

### 3.3. Relationship between GTPBP2 knockdown and Wnt/β-catenin signaling pathway

Western blot was used to detect the changes of Wnt/β-catenin signaling pathway-related proteins in A549, after knockdown GTPBP2. The results showed that β-catenin protein expression decreased and Axin protein expression increased (Figure 3H). This indicates that GTPBP2 may participate in the development of NSCLC through the Wnt/β-catenin signaling pathway.

## 4. Discussion

Lung cancer is one of the most common malignant tumors in the world, and the mortality rate ranks first among various malignant tumors. Data published showed that lung cancer accounted for 11.6% of all malignant tumors and 18.4% of deaths, still ranking first among malignant tumors 2. At present, recurrence and metastasis are still the main causes of patients' treatment failure and death, so finding effective targets has become one of the breakthroughs in the treatment of lung cancer.

GTPBP2 is a member of the G protein superfamily and has G protein kinase activity. The human GTPBP2 gene is located at 6p21.1 3. Mouse and human GTPBP2 have 92.8% and 99.1% similarity at the DNA and protein levels, respectively. This high degree of conservatism strongly suggests that GTPBP2 plays an important role among species 4. The human protein atlas shows that GTPBP2 is highly expressed in the esophagus and testis, but also in the lung, brain, and kidney. At present, in humans, research on GTPBP2 is limited to genetics 5. GTPBP2 allele-inactivating variants are associated with familial neurodevelopmental disorders and intellectual disabilities 6. This shows that GTPBP2 plays an important role in maintaining normal cell development.

Our results show that GTPBP2 is expressed in different degrees in NSCLC cells and is higher in A549 and H1299. GTPBP1 is the closest homologue of GTPBP2, and the two sequences are the most similar. Our experimental results show that the expression of GTPBP2 in NSCLC cancer tissues is higher than that of normal lung tissues, and its high expression is closed related to the TNM stage of lung cancer patients, lymph node metastasis and the poor prognosis of patients, indicating that GTPBP2 plays a role in the occurrence and development of NSCLC important role.

After GTPBP2 knockdown, there was no significant change in cell migration-related proteins RhoA and RhoB, RhoC expression decreased, and cell invasion-related protein MMP-9 expression decreased. RhoA, RhoB and RhoC, as members of the Rho family, play an important role in cell signal transduction. They can regulate the cellular skeleton, cell adhesion and cell transport, and regulate the invasion and metastasis of tumor cells 7-10. RhoA, as a single-molecule guanylate binding protein, plays an important role in the interaction between actin and myosin, and it can also be regulated by transcription factors such as HIF-1α/2α, c-myc, stat6 and microRNA 11. RhoB is a negative regulator of tumor, which can inhibit the biological functions of tumor invasion, migration and proliferation 12. RhoC has a small GTP-binding protein activity, which shows an activated state when it binds to GTP and an inactive state when it binds to GDP, and realizes signal transduction in this process 13. Our experimental results showed that after GTPBP2 knockdown, there was no significant change in RhoA and RhoB protein levels, but RhoC protein expression decreased. The specific mechanism of such changes in protein levels remains to be studied. Studies have shown that RhoC can up-regulate the expression level of MMP-9 and enhance the invasive activity of cells 14, which means that the expression of RhoC and MMP-9 are synchronized, and our experimental results also show that after knockdown of GTPBP2, RhoC and MMP-9 protein expression level decreased. In summary, it can be seen that cell migration and invasion are the result of the coordination of multiple proteins.

Knockdown of GTPBP2 resulted in decreased expression of cyclinD1, CDK4 and c-myc protein, and increased expression of P27. CDK4 can combine with cyclinD1 to form a complex, promote the transition from G1 phase to S phase, and promote cell proliferation 15, 16. CyclinD1 promotes the transition from G1 phase to S phase in the cell cycle, promotes DNA synthesis, and can overexpressed in various malignant tumors 17. P27 is a tumor suppressor gene that can inhibit the formation of CDK4/cyclinD1 complex 18. C-myc is a proto-oncogene that can regulate the transcription of target genes. Our experimental results show that after GTPBP2 silencing, the expression of cyclinD1 and CDK4 protein decreases, and the expression of P27 increases, which means that the formation of cyclinD1 and CDK4 complex is difficult to inhibit G1/S phase transition, thereby inhibiting the proliferation of tumor cells.

In addition, our study found that knock-down of GTPBP2, can make the expression of β-catenin decreased and Axin increased. Studies have shown that Wnt/β-catenin signaling pathway is closed related to tumorigenesis and development 19, 20. During normal cell development, Axin, as a negative regulator of the Wnt/β-catenin signaling pathway, can promote degradation of phosphorylated β-catenin and negatively regulate the Wnt/β-catenin signaling pathway, inhibiting tumorigenesis and development. The increased level of Axin can suppress degradation of phosphorylated β-catenin. Thus, the β-catenin accumulate in the cytoplasm and acts on transcription activators after entering the nucleus, causing malignant transformation of the tumor. Our results show that after knocking down GTPBP2, the level of Axin increases and the level of β-catenin decreases, that is, β-catenin may fail to enter the nucleus and activate transcriptional activators, which may lead to tumors. It's suggests that GTPBP2 may play a positive regulatory role in Wnt/β-catenin signaling pathway. Studies have shown that the C-terminus of GTPBP2 can directly interact with the MH1 domain of Smad1 to activate the BMP gene 21. After activation of BMP gene, it can lead to phosphorylation of downstream protein Smad1. Phosphorylated Smad1 forms a complex with Smad4, transfers to the nucleus, up-regulates the expression of nuclear transcription factors, inhibits the activity of epithelial marker E-cadherin, and promotes mesenchymal cell marker vimentin. Promote EMT, participate in the malignant transformation of tumor cells 22. The research suggests that GTPBP2 may play an important role in tumorigenesis by participating in the Wnt/β-catenin signaling pathway and EMT.

Therefore, we speculate that GTPBP2 may be a tumor suppressor gene for non-small cell lung cancer and participate in the development of non-small cell lung cancer. GTPBP2 may be a potential target for the treatment of non-small cell lung cancer and provide new ideas for the treatment of non-small cell lung cancer.

## Figures and Tables

**Table 1:** Antibodies used for western blotting in the present study.

Antibody name	Source	Catalog number	Host	Dilution
GTPBP2	Biorbyt	ER2180	Rabbit	1:2000
Axin1	Absin	Abs138784	Rabbit	1:2000
GAPDH	Wanlei Bio	WL01114	Rabbit	1:1000
RhoA	Proteintech	66733-1-Ig	Mouse	1:2000
RhoB	Proteintech	14326-1-AP	Rabbit	1:2000
RhoC	Proteintech	10632-1-AP	Rabbit	1:1000
MMP-9	Wanlei Bio	WL02141	Rabbit	1:500
c-Myc	Wanlei Bio	WL01781	Rabbit	1:1000
CyclinD1	Wanlei Bio	WL01435a	Rabbit	1:1000
P21	Wanlei Bio	WL03793	Rabbit	1:1500
CDK4	Wanlei Bio	WL02274	Rabbit	1:500
P27	Wanlei Bio	WL01769	Rabbit	1:500

**Table 2:** Association of GTPBP2 expression with clinical and pathological characteristics of NSCLC patients.

	Cases	Low expression	High expression	P
Age				
<60	46	15	31	0.129
≥60	66	31	35	
Gender				
Male	84	36	48	0.506
Female	28	10	18	
Histology type				
Squamous cancer	55	21	34	0.541
Adenocarcinoma	57	25	32	
Differentiation				
Well	32	17	15	0.146
Moderate-poor	80	29	51	
TNM stage				
I	54	31	23	0.005*
II+III	58	15	43	
Nodal status				
N0	60	31	29	0.014*
N1N2N3	52	15	37	
